# Assessment of Sustainability of Bio Treated Lignocellulose-Based Oleogels

**DOI:** 10.3390/polym13020267

**Published:** 2021-01-15

**Authors:** Carmen Fajardo, Alba Blánquez, Gabriela Domínguez, Antonio M. Borrero-López, Concepción Valencia, Manuel Hernández, María E. Arias, Juana Rodríguez

**Affiliations:** 1 Departamento de Biomedicina y Biotecnología, Universidad de Alcalá, 28805 Alcalá de Henares, Madrid, Spain; alba.blanquez@edu.uah.es (A.B.); gabriela.dominguez@edu.uah.es (G.D.); manuel.hernandez@uah.es (M.H.); enriqueta.arias@uah.es (M.E.A.); juana.rodriguez@uah.es (J.R.); 2 Departamento de Ingeniería Química, Campus de “El Carmen”, Universidad de Huelva, 21071 Huelva, Spain; am.borrero@diq.uhu.es (A.M.B.-L.); barragan@uhu.es (C.V.)

**Keywords:** lignocellulosic fermented residues, *Streptomyces*, bio-based oleogels, biodegradability, ecotoxicity, soil microbiome

## Abstract

The development of biological strategies to obtain new high-added value biopolymers from lignocellulosic biomass is a current challenge for scientific community. This study evaluates the biodegradability and ecotoxicity of new formulated oleogels obtained from fermented agricultural residues with *Streptomyces*, previously reported to show improved rheological and tribological characteristics compared to commercial mineral lubricants. Both new oleogels exhibited higher biodegradation rates than the commercial grease. Classical ecotoxicological bioassays using eukaryotic organisms (*Lactuca sativa*, *Caenorhabditis elegans*) showed that the toxic impact of the produced bio-lubricants was almost negligible and comparable to the commercial grease for the target organisms. In addition, high throughput molecular techniques using emerging next-generation DNA-sequencing technologies (NGS) were applied to study the structural changes of lubricant-exposed microbial populations of a standard soil. Results obtained showed that disposal of biomass-based lubricants in the soil environment did not substantially modify the structure and phylogenetic composition of the microbiome. These findings point out the feasibility and sustainability, in terms of biodegradability and eco-safety, of the new bio-lubricants in comparison with commercial mineral greases. This technology entails a promising biological strategy to replace fossil and non-renewable raw materials as well as to obtain useful biopolymers from agricultural residues with potential for large-scale applications.

## 1. Introduction

The depletion of petroleum resources, the increase in oil prices and the greater awareness of the current society opinion about preservation of the environment have impelled the scientific community to focus efforts on the use of natural alternative resources. The search for new biological strategies that allow not only the substitution of oil by renewable sources but also provide benefit to a variety of residues is a current challenge for biotechnologists. Thereby, researches have explored new renewable feedstock such as proteins, tree leaves, various seaweeds, vegetable oils, coffee pulp, paper mill sludge, lignocellulose and other agro-residues for the synthesis of a wide variety of products such as bio-plastic, bio-diesel, bio-lubricant, bio-adsorbent, bio-stimulants and bio-ethanol [1]. Particularly, the urgent need to diminish the climate alteration is pushing scientists to develop sustainable approaches/methodologies towards the transformation of lignocellulosic biomass into biofuels/bioenergy and range of value-added products [2,3,4].

Lignocellulose biomass has been used to obtain paper, biodegradable plastics, binders, feed, ceramics, pavements, chelating agents, production of boards, production of chemical compounds (phenols, catechol, vanillin, etc.) and even for medical uses (anti-inflammatory and anti-tumor activities) [5,6,7]. Biorefining of lignocellulosic biomass into these products is not only primarily linked to pollution prevention, but also offers sustainable development, involving the three pillars of sustainability: people, planet and profit (i.e., social, environmental and economic elements) [4].

However, its full exploitation, although attractive, is still far from being achieved. Even worse, the disposal of residual lignin from several industries is a major problem and, even now in some cases, its discharge to continental waters producing a large environmental impact is the only way out for this waste causing high pollution degree [8].

Previous studies carried out by our work group demonstrated that residual lignin obtained from lignocellulosic residues microbiologically pre-treated was a suitable raw material for the development of new products (bio-lubricants), contributing to the sustainable management of agricultural or industrial wastes [9]. In a further approach, the straight use of fermented lignocellulosic residues by streptomycetes to obtain oleogels was screened. For this, solid-state fermentation of two agricultural residues (wheat and barley straws) with different *Streptomyces* sp. strains was performed and once functionalized, the residues were used as thickeners to elaborate lubricant greases. The rheological and tribological analyses of the new formulations showed that these oleogels exhibited better properties and structures than some of the current industrial lubricants, highlighting their potential as substitutes of the commercial greases [10].

Lignin is a natural polymer, and although recalcitrant, it presents good biocompatibility and non-toxicity [11]. Accordingly, the new formulations produced are expected to be biodegradable to some extent, although the potential environmental associated risks to the eventual release (or disposal) of the lubricants could lead to unintended consequences [12]. Thus, research on oleogels biodegradation under soil environments, combined with evaluation of the environmental consequences of their exposure towards soil organisms, become a relevant aspect. 

Classical bioassays have been widely used to assess the impact of different chemicals on reference test organisms, and among them, *Caenorhabditis elegans* has been recognized as a useful bioindicator for toxicological studies in soil [13,14]. In addition, plants are also considered as key reference organisms in soil ecotoxicity assessment because they are crucial components of this ecosystem, maintaining its normal ecological function [15]. Nevertheless, for better understanding the impact of such chemicals on soil health and functionality, in addition to the classical tests, new relevant endpoints are increasingly considered. The growth and function of microorganisms, as well as the composition and diversity of the microbial community, can be severely affected by chemical substances that reaches the soil matrix [16]; thus, parameters such as the biodiversity and functionality of soil microbiota allow us to reliably monitor environmental impacts on contaminated soils.

This paper focusses on the ecotoxicity and biodegradability characteristics of two novel lignocellulose-based lubricants, compared to an industrial and commercially available grease. Classical ecotoxicological bioassays on terrestrial eukaryotic test organisms (*Lactuca sativa* and *C. elegans*) and high throughput molecular techniques using emerging next-generation DNA-sequencing technologies (NGS) were applied to study the impact of oleogels on soil biota and the structural modifications of lubricant-exposed microbial population of a standard soil.

## 2. Materials and Methods

### 2.1. Obtention and Chemical Composition of Oleogels

Bio-lubricants samples were obtained from Castor oil added of fermented straw (wheat and barley) with *Streptomyces* sp., and functionalized with 1,6-Hexamethylene diisocyanate (HMDI) (at a proportion 2:1), as previously reported. In brief, wheat straw and barely straw fermented with *Streptomyces* were directly blended with HMDI (1/2 cellulose pulp/HMDI weight ratio) and castor oil (85% or 90% *w*/*w*) under stirring at room temperature (~20 °C) for 24 h using an RW20 (IKA, Staufen, Germany) equipped with an anchor impeller at 70 rpm [10,17].

The obtained lignocellulose-based lubricants were named as follows: BL, obtained from fermented barley straw; WL, obtained from fermented wheat straw. Additionally, the commercial mineral grease Castrol Optipit^®^ (Castrol Limited, Landlord, BP, UK), containing lithium as thickener agent, was used for comparative purposes (named CL).

Elemental analyses (C, H, and N) of all lubricants were performed in triplicate using a LECO CHNS-932 analyser (LECO Instruments S.L, Tres Cantos, Spain) at the Centre of Chemical Analyses of the University of Alcalá.

### 2.2. Soil and Experimental Set Up

A standard and commercially available soil, Lufa 2.2 (LUFA Speyer, Speyer, Germany), was used to assess biodegradability and the lubricants-induced impact on exposed organisms (*C. elegans*, *L. sativa* and soil bacterial community). This soil is supplied as field fresh with active microbiota. According to the supplier, the physicochemical characteristics of this loamy-sand soil (USDA) were: total organic carbon, 1.77%; nitrogen, 0.17%; pH 5.5; cation exchange capacity, 10.1 meq/100 g; and water holding capacity, 41.8 g/100 g (all values refer to dry matter).

One kg per treatment of Lufa 2.2 soil (22% *w*/*w* humidity) was mixed with each tested sample (bio-lubricants and commercial grease) at a dose of 1% (*w*/*w*), while no oleogel-spiked soil was used as control (C).

### 2.3. Biodegradability of Lubricants: CO_2_ Evolution

The evaluation tests of lubricants’ biodegradability were carried out according to published protocols [18]. The test determines biodegradation over an incubation period, measuring CO_2_ evolution in the soil samples containing the assessed lubricants and compared with untreated soil. In brief, the testing vessels containing 50 g of each spiked soil (CL, BL and WL) and control soil (C) were incubated for 40 days at 28 °C and weighed periodically to maintain humidity (22% *w*/*w*). The carbon dioxide evolved by microbial respiration was captured in 10 mL sodium hydroxide solution (0.5 N), 5 mL barium chloride (0.5 M) was added, and the residual hydroxide was titrated with HCl 0.5 N. The carbon dioxide accumulated was calculated and represented versus the incubation time. All the experiments were run in triplicate.

### 2.4. Ecotoxicological Assays (C. elegans and L. sativa)

The ecotoxicity tests were performed at 0 days (t0) and 40 days post-incubation (t40). All the experiments were run in triplicate.

The phytotoxicity of commercial lubricant (CL) and bio-lubricants (BL and WL) was evaluated using *L. sativa* L. cultivar Trocadero as model organism and published protocols with minimal modifications [19]. In brief, *L. sativa* seeds were sterilized with ethanol 70% (*v*/*v*) for 10 min and thoroughly rinsed five times with sterilized distilled water. A total of 50 g of each lubricant-spiked soil were placed in a Petri dish and 10 seeds were sown per plate. Distilled water (4 mL) was added on the plates, being periodically replaced to maintain humidity. The Petri dishes were sealed with a cap with holes to allow air exchange and to avoid humidity loses, incubated in the dark for 48 h (room temperature), and afterwards under 16 h photoperiod. After 72 h, germinated seeds were counted. In accordance to Rede et al. [20], after seven days of exposure, we measured maximum root length to analyze plants development. Tests were considered valid if the germination of control test was ≥80% and the root length was up to 2 mm. 

Ecotoxicity assays in each analyzed soil were also performed using *C. elegans* as test organism and previous protocols [21,22]. In brief, the *C. elegans* wild type strain N2 was obtained from the *Caenorhabditis* Genetic Center (University of Minnesota, St. Paul, MN, USA), and maintained on nematode growth medium (NGM) plates seeded with *Escherichia coli* strain OP50 at 20 °C. To obtain nematodes at the first juvenile stage, chunks of agar starved plates with worms from stocks of dauer larvae were transferred to fresh NGM plates seeded with OP50 strain. After 3 days, age-synchronization of worms was achieved by washing them off the plates with K-medium (32 mM KCl, 52 mM NaCl, cholesterol 5 µg mL^−1^) and filtering through a 10 µm nylon net filter (Merck Millipore Ltd. Cork, IRL) which retained all but L1-L2 juvenile stage nematodes. The mean initial body length of 100 randomly selected L1 organisms was measured using a stereomicroscope (268 ± 2.2 µm, *s.e.m*).

The experiments were performed in 12-well microtiter plates (Nunclon Delta SI, Nunc, Roskilde, Denmark) in accordance with ISO 10872 [23]. Each well contained 0.5 mL of *E. coli* OP50 (2.5 × 10^10^ cells mL^−1^), five L1 nematodes, 0.2 mL K-medium and 0.3 g of each tested soil. After incubation for 96 h at 20 °C (in darkness), 0.5 mL of a Rose Bengal solution (0.4 g L^−1^) was added to each test well to stain the nematode cuticle and the experiment was stopped by heating (15 min, 80 °C). The endpoints reproduction, survival and growth were evaluated.

### 2.5. DNA Extraction, Metagenome Library Construction and Sequencing

Total bacterial community DNA was isolated from 0.5 g of each lubricant-spiked and control soils at 40 days post-incubation using the Power Soil DNA isolation kit (MoBio Laboratories, Inc., Carlsbad, CA, USA) and the manufacturer’s protocols. DNA libraries from each soil sample were prepared at the Genomics Unit of the Complutense University of Madrid (Spain) in accordance to published protocols [24]. Briefly, the V3-V4 region of the 16S rRNA gene was amplified with primers containing the 341F and 805R sequences and Illumina-specific adapters. In a second PCR amplification, two specific 8-nucleotide index and i5/i7 Illumina adapters were added to the previous amplicons. A library pool was prepared for sequencing by mixing equal amounts of the individual sample libraries and then subjected to electrophoresis. The bands containing the amplified fragments were excised, purified and sequenced (Illumina MiSeq, 2 × 300 reads) using the 600 cycle MiSeq Reagent Kit v.

The FASTQ files containing the sequencing reads were analyzed using the CLC Genomics Workbench version 11.0.1 (QIAGEN Aarhus A/S www.qiagenbioinformatics.com). Sequence data were trimmed (0.05 as a limit for quality scores, 2 as the maximum number of ambiguities) and further analyzed using the CLC Microbial Genomics Module version 4.0. Sequence reads were clustered and chimeric sequences detected using an identity of 97% as the Operational Taxonomic Unit (OTU) threshold. Reference OTU data used were downloaded from the Greengenes database v13_5 for 16S rRNA. The Shannon diversity index was calculated considering the assigned species. The β-diversity was determined by principal coordinate analysis (PCoA) based on weighed UniFrac distance.

### 2.6. Statistical Analyses

Statistical analyses were performed using the software package GraphPad Prism version 5.0 (Graph-Pad Software, San Diego, CA, USA). One-way analysis of variance followed by Tukey post-test were used for multiple comparisons to determine the significance of the differences between the groups.

The version available on the online WebMeV (Multiple Experiment Viewer) platform (http://mev.tm4.org) was used to perform hierarchical clustering. The table of OTUs generated by the CLC Microbial Genomics Module (QIAGEN) from each microbiome classified at class level was used as the input.

## 3. Results

### 3.1. Chemical Composition of Oleogels

The elemental analysis (%) of both lignocellulose-based oleogels BL and WL in comparison with that from the control lubricant (CL) was performed. The results obtained demonstrate that the carbon and hydrogen contents resulted higher in the commercial oleogel (CL) (84.46 and 12.25%, respectively) being both contents similar in oleogels BL and WL (71.48 and 71.76% of carbon content for BL and WL, respectively; 10.98 and 10.97% of hydrogen content for BL and WL, respectively). However, the nitrogen content of biomass-oleogels BL (2.92%) and WL (2.25%) was higher than in CL (1.48%). 

### 3.2. Biodegradability of Lubricants

**Figure 1:**
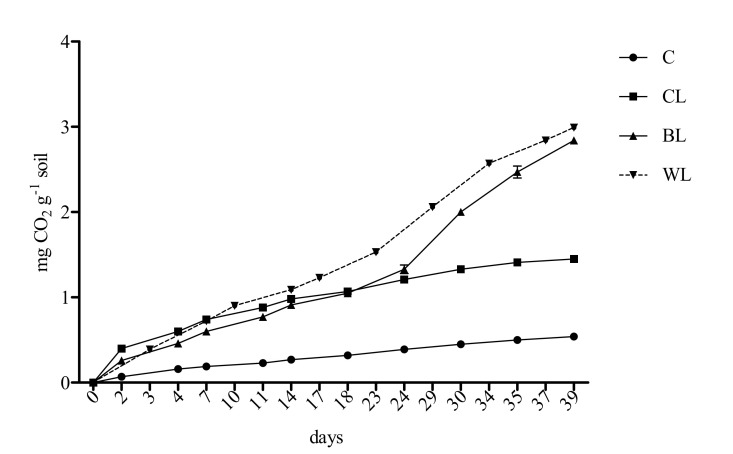
Accumulated CO_2_ production in the grease-spiked and control soils during the incubation period (n = 3, mean data ± s.e.m.).

The CO_2_ evolution in the soil samples was followed to determine the course of biodegradation of both lignocellulose-based lubricants (BL and WL) and commercial grease (CL) in comparison with the respirometric profile of the unamended soil sample (C). The results are shown in Figure 1.

Initially, soils spiked with both new formulated and commercial lubricants exhibited similar profiles and higher respiration than control sample (C). After 24 days, they evolved differentially, and the degradation rate of BL and WL samples clearly increased. Thus, the BL and WL soil samples showed higher CO_2_ production and, therefore, higher biodegradability than the soil amended with the commercial lubricant (CL), accounting for 2.84 and 2.91 mg CO_2_ accumulated g^−^^1^ soil after the incubation period, respectively. Accordingly, compared to control soil, BL and WL respiration rates were higher than 5-fold; related to the CL sample, a 2-fold increase was observed.

### 3.3. Toxicity of Lubricants on Selected Test Organisms

**Figure 2:**
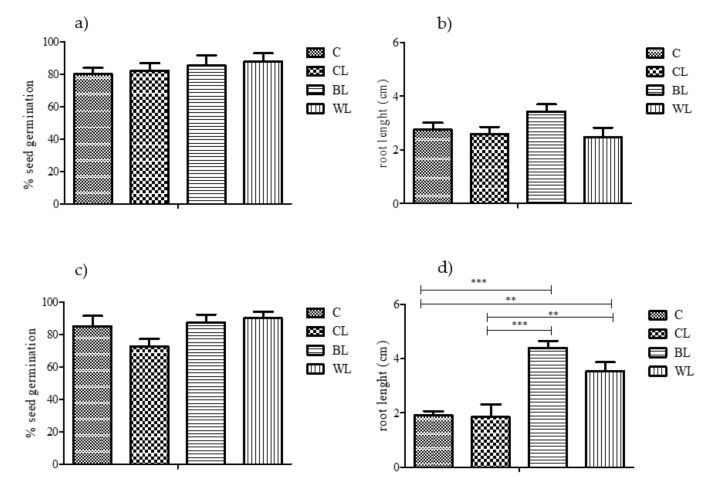
Seed germination percentage and root length of *L. sativa* in the control (C) and lubricant-spiked soil samples (CL, BL and WL): (**a**,**b**) data at t0; (**c**,**d**) data at t40 (n = 3, mean data ± s.e.m.). Significant differences at *p* < 0.01 (**) and *p* < 0.001 (***) are shown.

The effect of exposure to the tested lubricants in *L. sativa* seed germination and root elongation is shown in Figure 2. At t0, no significant effect on the considered endpoints, and thus no toxicity, was observed for the tested substances (*p* < 0.05). Similarly, after the incubation period (t40), the biodegradation process did not affect seed germination parameter, although an increase in root elongation was detected in BL and WL samples, compared to CL.

When C. elegans was exposed to the lubricant-spiked soils (CL, BL and WL) at t0, two endpoints (survival and reproduction) did not show statistically significant differences relative to the control soil (C) (Table 1). Only growth parameter was slightly decreased in worms exposed to BL and WL samples. After 40 days we found a similar pattern, and only growth parameter decreased in CL, BL and WL spiked soil samples compared to control.

### 3.4. Soil Microbiome Analyses

High-throughput sequencing of 16S rRNA gene was performed to assess the impact of the tested lubricants, both lignocellulose-based and mineral, on the biodiversity and phylogenetic composition of soil microbiomes. The mean number and percentage of OTUs classified at the phylum, class, genus, and species levels in each analyzed microbiome are shown in Table 2.

To compare the species richness among the tested samples, the rarefaction curves were generated (97% cut-off). The samples exhibited similar trends and all curves tended to reach saturation; thus, we can assume that the sampling representativeness was high enough to detect most sequence types (Figure S1, Supplementary Information). The Shannon index suggested a similar diversity among the assessed soil samples, although alpha diversity in BL microbiome was lower than in control sample (C) (Table 2).

**Figure 3:**
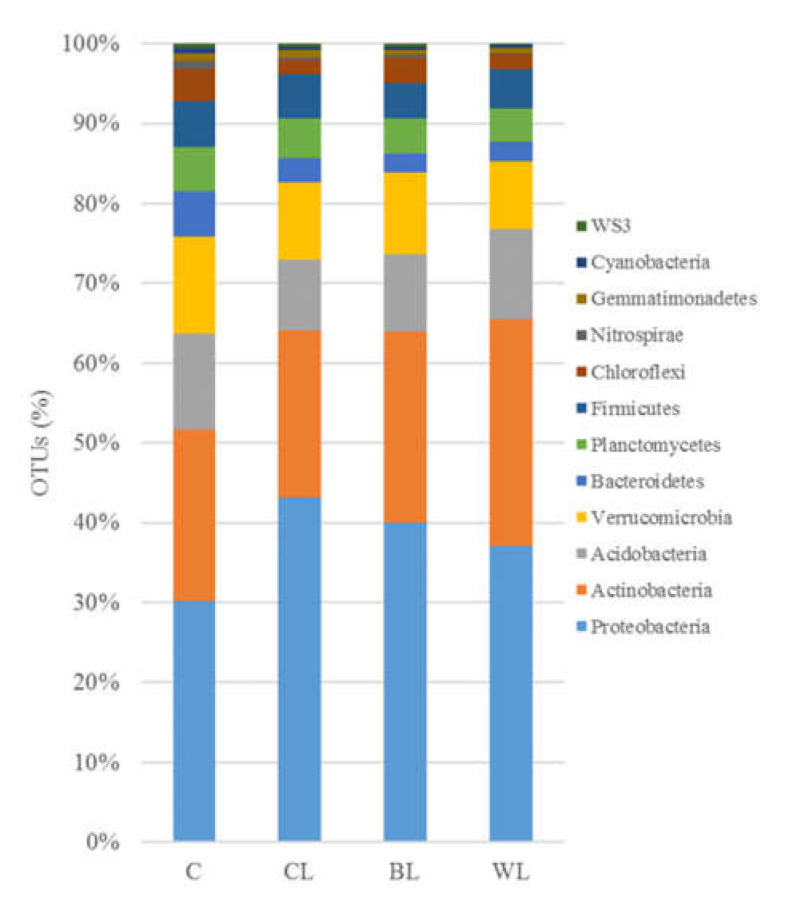
Relative abundances of phyla in the control and grease-spiked soil microbiomes (mean values, n = 3). Relative abundances are based on the proportional frequencies of OTUs that could be classified.

The total number of phyla ranged from 31 in C soil to 24 in WL sample. Only 12 phyla showed a relative abundance >0.5% in all the samples, where *Proteobacteria, Actinobacteria, Acidobacteria* and *Verrucomicrobia* represented >75% of all the identified sequences (Figure 3).

**Figure 4:**
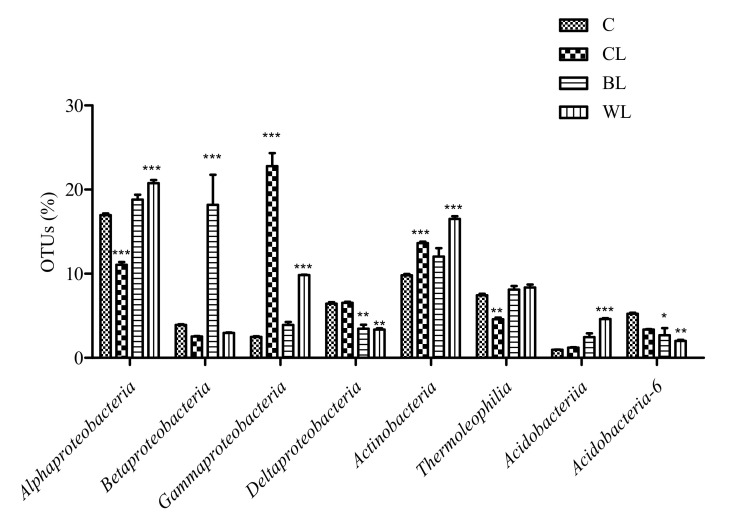
Most abundant identified classes in control soil and lubricant-spiked soils after 40 days of exposure (n = 3, mean values ± s.e.m.). Significant differences at *p* < 0.05 (*), *p* < 0.01 (**) and *p* < 0.001 (***) with respect to control soil are shown.

At the phylum level, microbiomes of all soils supplemented with lubricants (commercial and lignocellulose-based) showed scarce structural shifts compared to control soil (*p* < 0.05). The samples BL, WL and, particularly, CL were enriched in Proteobacteria (Figure 3). The observed higher abundance of reads belonging to this phylum was due to the increase of the class γ-Proteobacteria, mostly in CL, α-Proteobacteria in WL, or β-Proteobacteria in BL sample (Figure 4).

The Actinobacteria phylum was significantly overrepresented in the sample WL (28.3%), while it showed decreased abundance in OTUs affiliated to Verrucomicrobia phylum, compared to control soil (C) (Figure 3). At the class level, Actinobacteria taxon was increased within microbial communities of CL and WL soil samples (13.6 and 16.5%, respectively), although a decreased number in the OTUs affiliated with Thermoleophilia class was detected in CL (Figure 4).

When compared the microbiomes of the different lubricant-spiked soils, we found some class-specific responses (*p* < 0.05). Lignocellulose-based oleogels induced increase in the bacterial population belonging to α-Proteobacteria and Thermoleophilia classes, whereas CL sample was enriched in OTUs affiliated to γ- and δ-Proteobacteria. The microbiomes of BL and WL samples followed a similar pattern, and their taxonomical profiles only differed in the Actinobacteria, β- and γ-Proteobacteria population.

**Figure 5:**
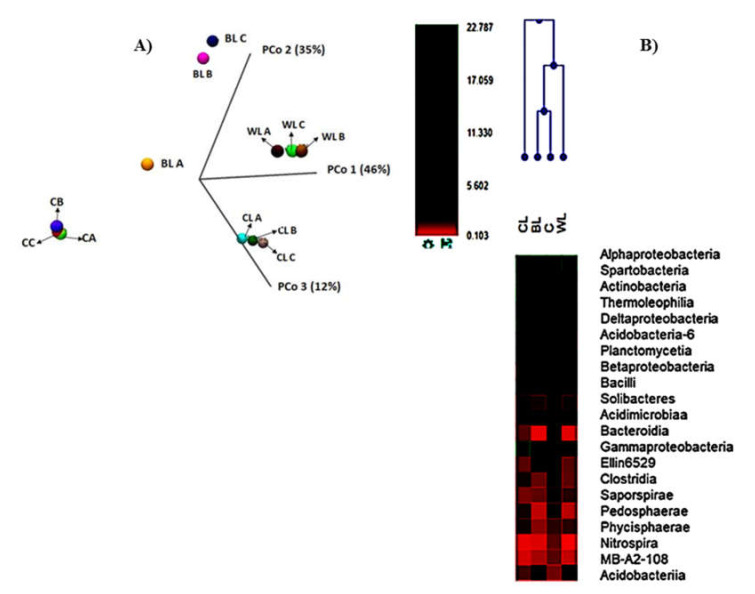
Principal coordinate analysis (PCoA) Euclidian beta diversity of the soil metagenomes (**A**), heat map and dendrogram (**B**) of the most abundant bacterial classes in the microbial community of soil samples.

The hierarchical clustering and PCA analyses supported the differences previously described among the assessed samples in terms of the phylogenetic structure (Figure 5). Soil microbiomes at the class level appeared grouped into three different clusters: CL sample clustered separate from control soil and samples amended with the lignocellulose-based lubricants.

## 4. Discussion

The huge amount of base stock oils and finished lubricants worldwide used makes these products likely to end up and accumulate in ecosystems, through production and distribution processes, usage or disposal after their utilization [25]. Biodegradability is a crucial parameter to determine the environmental risk associated with those chemicals when deployed in the environment. If the lubricant, due to its hydrophobic nature, remains adsorbed by the soil particles and is biodegradable, it could be transformed by the soil’s microorganisms, decreasing environmental damage [26]. Therefore, high biodegradability implies a reduced tendency to bio-accumulate or to persist in the environment [27]. 

In this study, we have evaluated the biodegradability of two lignocellulose-based lubricants and compared the same feature of a commercial one widely employed as lubricant. Previously, we have demonstrated that the bio-based oleogels, obtained after the biological treatment, exhibited favorable viscoelastic responses and viscosity values. Furthermore, the friction coefficient and dimensions of wear scars measured in a tribological contact were comparable to, or even lower than, those found with commercial lubricating greases [10].

The main components of the lignocellulose-based oleogels are natural compounds, and thus, they were expected to be potentially biodegradable; this point was further demonstrated in this study, even when the new greases also contained HMDI. Here, higher biodegradation rates of the new formulated bio-lubricants with respect to the commercial grease were obtained within 40 days of incubation. These results are in accordance with previous studies, where bio-lubricants (vegetable-based oils) showed ultimate biodegradability higher than a fluid of mineral origin, especially in a soil environment [26]. Both tested lignocellulose-based oleogels showed very similar degradation kinetics and differed from that of the mineral lubricant. Differences in the biodegradability of the assessed products over time could be mainly ascribed to the complex chemical mixture (including lithium) contained in the commercial grease. According to their elemental composition, soil amendment with all tested greases might imply an initial increase in the organic matter content available for the aerobic heterotrophic microorganisms. Consequently, in the first weeks, a similar and enhanced respirometric profile was recorded in the spiked soil samples compared to the unamended control soil. However, the organic C consumption would lead to a release of lithium to the soil environment treated with the commercial lubricant, which could delay the biodegradation process. After this point, significant differences among bio-lubricants and commercial grease were evident. The obtained results highlighted that lignocellulose-based oleogels were biodegraded at higher rates than the industrial lubricant, providing them key valuable features to be considered as eco-safety products.

When considering complex chemical mixtures such as commercial lubricant agents, where the complete composition is often unknown, chemical analyses might provide partial information on the potential hazards for the environment related to their inadequate management. However, biological-based approaches are useful strategies to achieve this goal [28]. In this study, we conducted a set of bioassays to test the potential ecotoxicological effects of bio-lubricants, using the nematode *C. elegans* and the plant *L. sativa* as test organisms. A decrease in the growth of *C. elegans* was detected in BL and WL samples at t0, although at t40, all three spiked soils (CL, BL and WL) exhibited similar toxicity towards exposed worms. Although previous studies reported ecotoxicity of mineral and semi-synthetic commercial lubricants [29], our results from the bioassay using *L. sativa* showed no effect of tested lubricants at t0, either in the germination rates or in root length of plants; thus, lignocellulose-based oleogels did not produce toxic impact on the plant. Even more, after the biodegradation process has occurred, an increase in root length of exposed plants was found in the soils amended with the novel lubricants. It is well known that microbial transformation of lignocellulosic biomass leads to the release of sub products (i.e., aromatic acids) which could in turn be incorporated into soil humic fractions [30]. The observed increase in the root length after 40 days of soil exposure to the assessed bio-lubricants could be a consequence of the organic matter soil enrichment.

Therefore, the overall results showed that the toxic impact of the produced bio-lubricants was almost negligible for *L. sativa* in comparison with the control soil (even a positive effect on the root length once the biodegradation occurred was observed). The effect on *C. elegans* was comparable to the commercial grease for this eukaryotic target organism. Microorganisms are known to play key roles in soil and other ecosystems but several stressors, such as chemicals released to the environment, can adversely affect them. In this study, we assessed the lubricant-induced perturbation on the microbial community composition in order to know their potential impact on soil ecosystem functions [16]. The application of high-resolution molecular techniques (NGS) allowed generation of metagenomic profiles of the soil microbiomes, and identification of shifts on microbial diversity in response to chemical-induced stress.

We found that addition of lubricants at the assessed concentration did not induce remarkable impact on the soil microbiome: phylogenetic profile of control soil did not dramatically differ from treated soils after the biodegradation process has occurred. *Proteobacteria* was particularly abundant in the grease-spiked soils compared to the untreated sample, which is consistent with previous studies that have reported *Proteobacteria* as major phylum in soils added of used oil lubricants or hydrocarbon-containing soils [31]. In this context, CL soil showed an increased percentage of γ*-Proteobacteria*, which includes many hydrocarbon-degrading genera as *Pseudomonas*.

Particularly, our results showed that disposal of lignocellulose-based lubricants in the soil environment did not disturbed the structure and phylogenetic composition of the microbiome. Although some phylum- and class-specific responses linked to the grease amendments were highlighted, the obtained results revealed that after the biodegradation process, the microbiome of soils with lignocellulose-based substances added exhibited higher similarity to the control soil than the sample treated with the commercial grease.

## 5. Conclusions

This study demonstrates that lignocellulose-based oleogels exhibited, according to most assays performed, improved or at least comparable essential features for environmental sustainability. So far, biodegradability and eco-safety of these oleogels get better when compared with a commercial grease. In addition, the biodegradation process that takes place in soil entailed no risk for the surrounding soil biota; no marked disturbance was found on bacterial phylogenetic profile or null phytotoxic impact and only slightly over *C. elegans*. Therefore, this study highlights the great potential of using lignocellulose-based oleogels to replace commercial greases providing an innovative approach to obtain biopolymers through the upgrading of agricultural residues without negative impact on the environment. Both achievements fit well the increasing social demand for environmentally friendly technologies and circular economy requirements.

## Figures and Tables

**Table 1:** Growth, reproduction and survival ecotoxicological endpoints of *C. elegans* in the lubricant-spiked and control soils, at 0 and 40 days post-incubation. Values are given as mean ± s.e.m. (n = 12).

SAMPLE	Growth (µm)		Reproduction (Offsprings/Adult)		Survival (Worms)	
	t0	t40	t0	t40	t0	t40
C	1114 ± 11	1061 ± 11	113 ± 11	84 ± 3.9	8.8 ± 0.5	9.0 ± 0.8
CL	1121 ± 10	1021 ± 9.4 *	136 ± 24	84 ± 5.1	8.7 ± 0.7	9.9 ± 0.7
BL	1050 ± 8.2 ***	881 ± 9.6 ***	129 ± 7.3	63 ± 6.9	9.1 ± 0.3	9.3 ± 0.7
WL	1017 ± 7.2 ***	909 ± 10 ***	108 ± 7.9	67 ± 5.7	8.6 ± 0.4	9.3 ± 0.4

*: Significant differences (*p* < 0.05) between control and treatment. ***: Significant differences (*p* < 0.001) between control and treatment.

**Table 2:** Data obtained after bacterial community DNA sequencing and classification of Operational Taxonomic Units (OTUs) according to the CLC Microbial Genomics Module and the Greengenes database (n = 3, mean data ± *s.e.m*.).

SAMPLE	Nº OTUs	%Classified to Phylum	%Classified to Class	%Classified to Genus	%Classified to Species	Shannon Index
C	5383 ± 285	100 ± 0.00	100 ± 0.00	35.17 ± 0.45	1.42 ± 0.03	3.3424 ± 0.034
CL	4278 ± 337 *	100 ± 0.00	100 ± 0.00	29.23 ± 1.08	2.30 ± 0.04	2.7394 ± 0.040
BL	3660 ± 93 **	100 ± 0.00	100 ± 0.00	46.75 ± 4.34	13.09 ± 5.53	1.7051 ± 0.907 *
WL	3921 ± 70 **	100 ± 0.00	100 ± 0.00	39.74 ± 0.38	2.66 ± 0.10	3.0015 ± 0.037

*: Significant differences (*p* < 0.05) between control and treatment. ** Significant differences (*p* < 0.01) between control and treatment.

## Data Availability

Data sharing is not applicable to this article.

## Supplementary Materials

The following are available online at https://www.mdpi.com/2073-4360/13/2/267/s1, Figure S1: title: Rarefaction curves (alpha-diversity) for the analyzed microbiomes at the 97% similarity level.
