# Supplementary material for: Assessment of Sustainability of Bio Treated Lignocellulose-Based Oleogels
Source: Polymers (Basel). 2021 Jan 15;13(2):267. doi: 10.3390/polym13020267 (PMC7829808; doi:10.3390/polym13020267)
Supplement: Supplementary file 1 [file polymers-13-00267-s001.pdf]

SUPPLEMENTARY INFORMATION

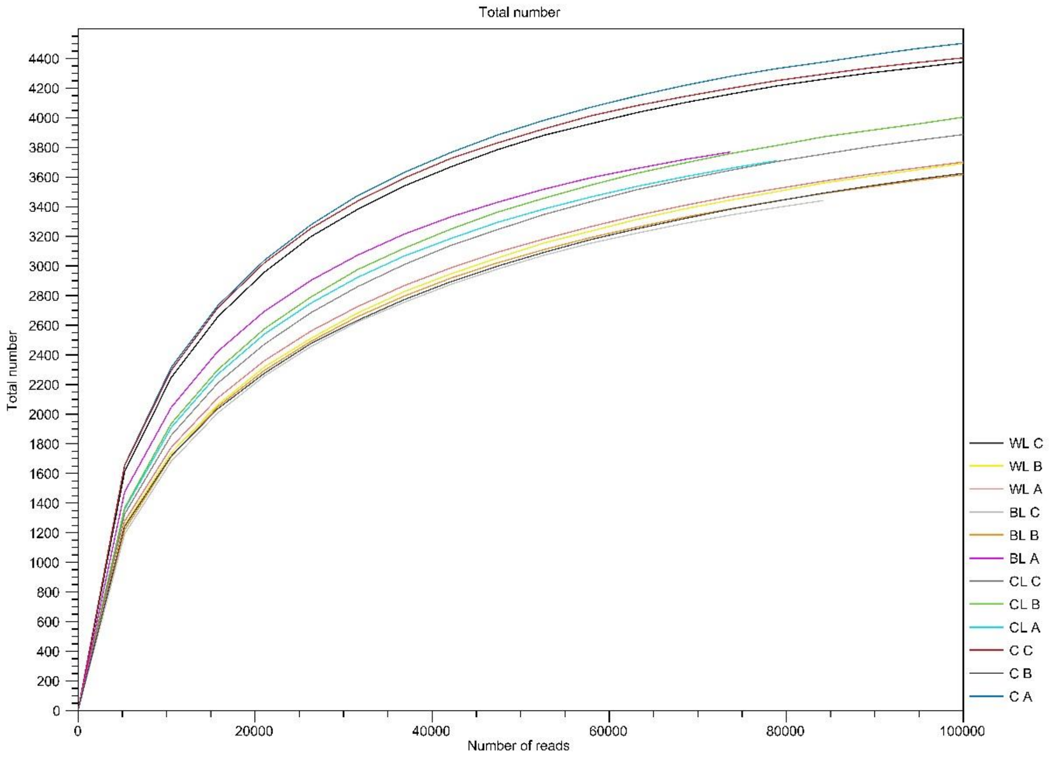

Figure S1. Rarefaction curves (alpha-diversity) for the analyzed microbiomes at the 97% similarity level.
